# Combination of Perindopril Erbumine and Huangqi-Danshen Decoction Protects Against Chronic Kidney Disease via Sirtuin3/Mitochondrial Dynamics Pathway

**DOI:** 10.1155/2022/5812105

**Published:** 2022-05-21

**Authors:** Xian Wei, Yuzhi Wang, Jiali Weng, Yunlan Lao, Ruyu Deng, Jiandong Lu, Shudong Yang, Xinhui Liu

**Affiliations:** ^1^Department of Nephrology, Shenzhen Traditional Chinese Medicine Hospital, Guangzhou University of Chinese Medicine, Shenzhen 518000, China; ^2^The Fourth Clinical Medical College, Guangzhou University of Chinese Medicine, Shenzhen 518000, Guangdong, China; ^3^Shenzhen Traditional Chinese Medicine Hospital, Nanjing University of Chinese Medicine, Shenzhen 518000, Guangdong, China

## Abstract

**Background:**

Chronic kidney disease (CKD) is a major public health problem worldwide. Treatment with renin-angiotensin system inhibitors can achieve only partial efficacy on renal function decline and renal fibrosis in CKD patients. Huangqi-Danshen decoction (HDD) is a basic Chinese herbal pair which is commonly used to treat CKD with good efficacy.

**Objectives:**

The current study aimed to investigate the effect of perindopril erbumine (PE), an angiotensin-converting enzyme inhibitor, combined with HDD on adenine-induced CKD rat model and explore the possible mechanism from Sirtuin3/mitochondrial dynamics pathway

**Method:**

CKD rat model was established by feeding of 0.75% w/w adenine containing diet for 3 weeks. At the same time, the treatment groups were given PE (0.42 mg/kg/d) or HDD (4.7 g/kg/d) or PE combined with HDD by gavage for 4 weeks. Renal function was evaluated by the levels of serum creatinine (Scr) and blood urea nitrogen (BUN). The renal pathological injury was observed by periodic acid-Schiff (PAS) and Masson's trichrome staining. Proteins expression was determined by Western blot analysis. Mitochondrial morphology was observed by transmission electron microscopy.

**Results:**

PE in combination with HDD significantly improved renal function, reduced tubular injury and interstitial fibrosis in adenine-induced CKD rats. Moreover, PE + HDD treatment mainly activated the Sirtuin3 expression level. In addition, PE + HDD exhibited bidirectional regulation on mitochondrial dynamics by suppressing mitochondrial fission protein dynaminrelated protein 1 expression and elevating mitochondrial fusion protein optic atrophy 1 expression, resulted in restraint of mitochondrial fragmentation.

**Conclusion:**

The combination of PE and HDD attenuated adenine-induced CKD in rats, which was possibly associated with Sirtuin3/mitochondrial dynamics pathway.

## 1. Introduction

Chronic kidney disease (CKD) is a major public health problem worldwide [1], and approximately 10% of global adults suffer from CKD [2]. According to the cause-specific mortality investigation, CKD was estimated to become the fifth leading cause of death by 2040 [3]. Treatment with renin-angiotensin system inhibitors (RASIs), such as angiotensin-converting enzyme inhibitors (ACEIs) and angiotensin II receptor blockers (ARBs), is the standard therapeutic strategy for patients with CKD [4]. However, the RASIs have partially triumphant as these compounds just delay, rather than prevent the progression to end-stage renal disease (ESRD) [5]. In addition, the incidence of ESRD remained gradually increased over decades. Therefore, further comprehensive exploration of safe and effective treatment applied for CKD is necessary.

As we know, traditional Chinese medicine (TCM) owned a long history dating back thousands of years for clinical therapy of diverse diseases [6]. Combination therapy of TCM with standard western medicine has been considered as an effective and complementary approach for patients with CKD [7,8]. Huangqi-Danshen decoction (HDD) is composed of Astragali Radix (Huang-qi) and Salviae Miltiorrhizae Radix et Rhizoma (Dan-shen). HDD is widely used as a basic herbal pair in CKD treatment with good efficacy. Our previous studies demonstrated that HDD could preserve renal function and inhibit renal fibrosis in CKD animal models [9,10]. However, the therapeutic efficacy and mechanisms of HDD in combination with RASI in CKD have not yet been fully elucidated.

Kidney is one of the most mitochondria-rich organs besides heart and liver [11]. Cumulative evidence indicated that the mitochondrial dynamics are closely linked to renal diseases [12]. Mitochondrial dynamics balance is achieved by continuous mitochondrial fission and fusion, and has the function of maintaining mitochondrial shape, number and quality control [13]. Under stress or injury conditions, imbalanced mitochondrial dynamics lead to mitochondrial fragmentation and mitochondrial dysfunction. Accumulating evidence indicated that Sirtuin3 played a vital role in mitochondrial dynamics modulation, closely associated with cancer, neurodegenerative and autoimmune diseases [14–16]. Decreased Sirtuin3 expression was associated with mitochondrial dynamics imbalance in the cisplatin-induced acute kidney injury (AKI) [17] and renal ischemia-reperfusion injury animal models [18]. However, the role of Sirtuin3/mitochondrial dynamics pathway in CKD progression is merely investigated.

In the present study, we explored the therapeutic efficacy of perindopril erbumine (PE), an ACEI, combined with HDD in the treatment of CKD, and investigated the underlying mechanism in terms of Sirtuin3/mitochondrial dynamics pathway by using adenine-induced CKD rat model.

## 2. Materials and Methods

### 2.1. Drugs

HDD is composed of Astragali Radix [roots of *Astragalus* membranaceus (Fisch). Bge. Var. Mongholicus (Bge). Hsiao] and Salviae Miltiorrhizae Radix et Rhizoma (roots and rhizomes of Salvia miltiorrhiza Bge). The herbs were weighted according to the ratio of 2 : 1 (w/w), boiled twice in 8 × ddH_2_O (w/v) for 1h per time. The concentrate liquid was centrifuged, and the supernatant was freeze-dried and stored at -80°C. Before treatment, we redissolved the powder with ddH_2_O at room temperature to get the HDD extract. The quality control of the decoction was conducted via high-performance liquid chromatography-mass spectrometry (HPLC-MS) analysis as described in our previous study [9]. The PE (HY–B0130 A) was purchased from MCE (MedChemExpress, Monmouth Junction, NJ, USA).

### 2.2. Animal Models

The animal experiment protocols were approved by the Ethics Committee of Guangzhou University of Chinese Medicine. Male 8-weeks-old Spraque-Dawley (SD) rats (*n* = 30) were purchased from Guangdong Medical Laboratory Animal Center (Foshan, China) weighting 220–260g. All animals were maintained in 12 h light/dark cycles and free access to water and food, at a constant temperature (22–25°C) and humidity (40–70%). After 7 days of adaptive feeding, all rats were randomly allocated into five groups: the control group (*n* = 6), the CKD model group (*n* = 6), the PE treatment group with CKD (*n* = 6), the HDD treatment group with CKD (*n* = 6), and the PE + HDD treatment group with CKD (*n* = 6). CKD was induced in rats by feeding adenine (Sigma-Aldrich, St Louis, MO, USA) in feed at a concentration of 0.75% w/w for 3 weeks. Treatment group rats were received 0.42 mg/kg/d dose of PE or (and) 4.7 g/kg/d dose of HDD extract orally for 4 weeks with simultaneous adenine feeding. At the end of the experiment, all rats were anesthetized, and blood samples were obtained from abdominal aorta. The rats were euthanized by cervical dislocation without regaining consciousness. Kidneys were removed and preserved for histological analysis and Western blot analysis.

### 2.3. Biochemical Analysis

Serum creatinine (Scr) and blood urea nitrogen (BUN) were measured by creatinine serum detection kit and BUN detection kit (StressMarq Biosciences, British Columbia, Canada). All procedures were carried out according to the manufacturer's protocol.

### 2.4. Histological Analysis

The excised kidney tissues obtained from the sacrificed rats were fixed in 4% paraformaldehyde at 4°C overnight, then dehydrated using a graded ethanol washing process and embedded in paraffin. The embedded kidney tissues were cut into 4 mm and stained with periodic acid-Schiff (PAS) and Masson's trichrome stains for morphological assessment. The tubular injury score was performed in PAS staining and were based on tubular epithelial cells atrophy, shedding, and tubular dilation. The score system was: 0 = no tubular injury; 1 = <10%; 2 = 10–25%; 3 = 26–50%; 4 = 51–75%; and 5 = >75% tubular injury [19]. Tubulointerstitial fibrosis in Masson staining was evaluated by collagen volume fraction (CVF) using the ImageJ software (National Institutes of Health, USA). CVF was calculated as follows: collagen area/total area × 100% [20]. One microscopic field (200×) of each rat and 6 rats in each group were performed quantitative analyses in a blinded manner.

### 2.5. Western Blot Analysis

Total proteins isolated from the kidney cortexes were homogenized in lysis buffer and centrifuged at 12000 rpm for 10 min at 4°C. The supernatant, which was used to extract the protein sample, was assessed using the Bradford protein detection system (assay). Proteins were then denatured with an equal amount of 4 × SDS sample buffer at 100°C for 10 minutes. After preprocessing, sample proteins were separated by 7% or 10% SDS-PAGE gels and transferred to nitrocellulose membrane (Millipore, Billerica, MA, USA). Next, the strips were blocked for 1h with 5% skimmed milk dissolved in Tris-buffered saline with 0.1% Tween 20 (TBST) for 1h at room temperature. The blots were then incubated with primary antibodies overnight at 4 C. Primary antibodies included Sirtuin 3 (1 : 500; Proteintech, no. 10099-1-AP), dynaminrelated protein 1 (Drp-1, 1 : 1000; Cell Signaling Technology, no. 8570), optic atrophy 1 (OPA-1, 1 : 2000; Proteintech, no. 27733-1-AP), fibronectin (FN, 1 : 250; Abcam, no. 2413), type IV collagen (Col-IV, 1 : 250; Abcam, no. 6586), *α*-smooth muscle actin (*α*-SMA, 1 : 1000; sigma, no. A2547), glyceraldehyde-3-phosphate dehydrogenase (GAPDH, 1 : 5000; Proteintech, no. 60004-1-lg). Binding of primary antibodies was followed by incubation for 1h at room temperature with the secondary horseradish peroxidase-conjugated IgG (Life Technologies, Carlsbad, CA, USA). The bands were visualized using ECL chemiluminescence (Millipore, Billerica, MA, USA) and a ChemiDoc MP Imaging System (Bio-Rad Laboratories, Hercules, CA, USA).

### 2.6. Transmission Electron Microscopy (TEM)

Freshly kidney cortexes of all groups were cut into 1 mm^3^ pieces using for TEM kidney samples in cold 2.5% glutaraldehyde solution. The samples were applied to routine dehydration, osmosis, then embedded in epoxy resin. Ultrathin sections were harvested (EM UC7, Leica, Wetzlar, Germany), and renal mitochondria were observed using a transmission electron microscopy (HT7700, Hitachi, Tokyo, Japan).

### 2.7. Statistical Analysis

The data were performed with GraphPad Prism (version 7.0, GraphPad software, San Diego, Calif, USA) and were presented as mean ± SEM for graphical representation. The statistical results were conducted with Tukey's multiple comparisons test in ANOVA, and *P* < 0.05  were regarded statistically significant.

## 3. Results

### 3.1. Effects of PE and HDD on Renal Function in CKD Rats

**Figure 1:**
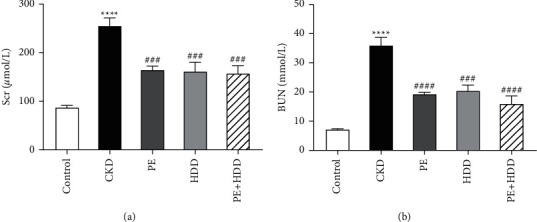
Effects of PE and HDD on renal function in CKD rats. The levels of Scr (a) and BUN (b) in all groups. All data are expressed as the means ± SEM, *n* = 4–6 rats per group ( ^*∗*^ ^*∗*^ ^*∗*^ ^*∗*^*P* < 0.0001) compared with the control group; ^###^*P* < 0.001, ^####^*P* < 0.0001 compared with the CKD group).

As shown in Figure 1, the levels of Scr and BUN were both raised in all CKD rats when compared to the control group (*P* < 0.0001). Compared with the CKD group, Scr and BUN levels were significantly suppressed by PE or HDD treatment. Moreover, the combination group obviously reduced the Scr (*P* < 0.001) and BUN (*P* < 0.0001) levels respectively. These results indicated the CKD model was established successfully and the levels of Scr and BUN were significantly declined in all three treatment groups.

### 3.2. Effects of PE and HDD on Renal Pathological Injury

**Figure 2:**
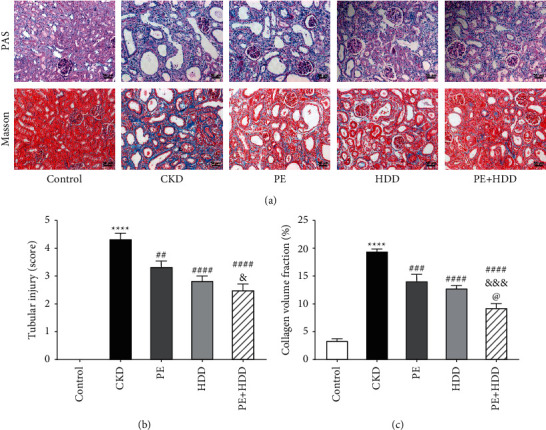
Effects of PE and HDD on renal pathological injury. (a), Renal structure in PAS and Masson staining of Control, CKD, PE, HDD and the combination treatment group. (b), Quantitative analysis of renal tubular injury score in all groups. (c), Quantification of collagen volume fraction (collagen area/total area × 100%) in all groups. All data are expressed as the means ± SEM, *n* = 6 rats per group (^*∗∗∗∗*^*P* < 0.0001 compared with the control group; ^##^*P* < 0.01, ^###^*P* < 0.001, ^####^P  <  0.0001 compared with the CKD group; ^&^*P* < 0.05, ^&&&^*P* < 0.001 compared with the PE treatment group; ^@^*P* < 0.05 compared with the HDD treatment group).

Tubulointerstitial injury is considered to be the major renal injury pathology in all forms of CKD [21], featured as tubular collapse, tubular atrophy and interstitial fibrosis [22]. As shown in Figure 2(a) and 2(b), PAS staining of CKD group exhibited massive tubular epithelial cells atrophy, shedding, and tubular dilation compared with control group (*P* < 0.0001). Furthermore, PE + HDD group exhibited less tubular injury compared with PE group (*P* < 0.05). Consistently, the Masson staining showed significant collagen deposition and tubulointerstitial fibrosis in the CKD group (Figure 2(a) and 2(c)). As to treated groups, PE in combination with HDD treatment group displayed a marked reduction of tubulointerstitial fibrosis compared to HDD treatment (*P* < 0.05) and PE treatment (*P* < 0.001).

### 3.3. Effects of PE and HDD on Renal Fibrosis in CKD Rats

**Figure 3:**
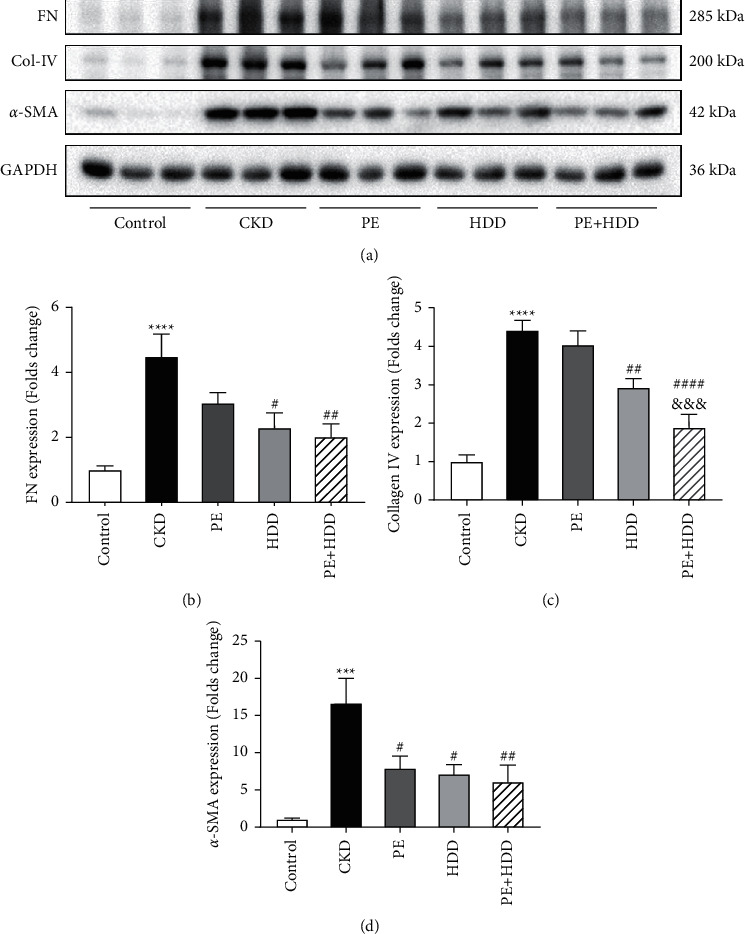
Effects of PE and HDD on renal fibrosis in CKD rats. (a), Representative Western blot images of FN, Col-IV and *α*-SMA. (b)-(d), Quantitative analysis of the protein expression of FN, Col-IV and *α*-SMA, respectively. The data are expressed as the means ± SEM, *n* = 6 rats per group ^*∗∗∗∗*^*p* < 0.0001), ^*∗∗∗*^*P* < 0.001 compared with the control group; ^#^*P* < 0.05, ^##^*P* < 0.01, ^####^*P* < 0.0001 compared with the CKD group; ^&&&^*P* < 0.001 compared with the PE treatment group).

The increased expressions of FN, Col-IV and *α*-SMA are crucial signals of renal tubulointerstitial fibrosis in the CKD progression.  Figure 3 displayed the anti-fibrotic effects of the three treatments. Compared with the CKD group, the expression of *α*-SMA was significantly downregulated by PE or HDD (*P* < 0.05). As shown in Figure 3(b)–3(c), 3 weeks of adenine-administration resulted in a significantly increased expression of Col-IV and FN (*P* < 0.0001). HDD attenuated this trend, and amplified this anti-fibrotic effect in combined with the PE treatment (*P* < 0.01). In addition, the combination of the two treatments exhibited more inhibitory effect on Col-IV expression than PE treatment alone (*P* < 0.001). However, the PE treatment did not significantly reduced Col-IV and FN expression. These results indicated the inhibitory effects of PE combined with HDD treatment on renal fibrosis.

### 3.4. Effects of PE and HDD on Sirtuin3 Expression in CKD Rats

**Figure 4:**
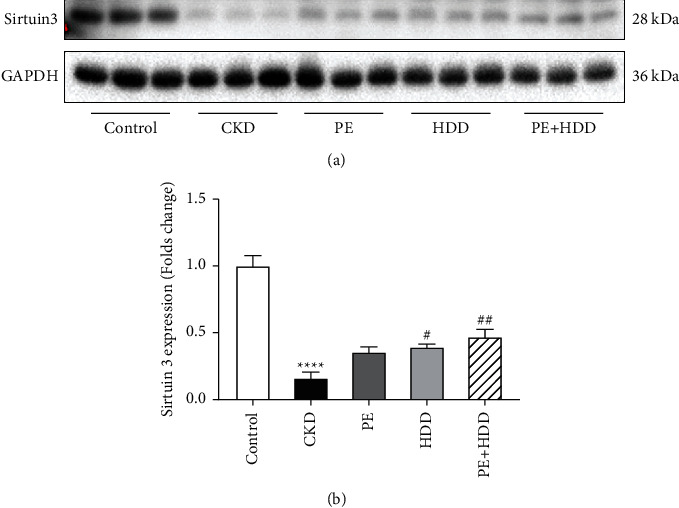
Effects of PE and HDD on Sirtuin3 expression in CKD rats. (a), Representative Western blot images of Sirtuin3. (b), Quantitative analysis of protein expression of Sirtuin3. The data are expressed as the means ± SEM, *n* = 6 rats per group (^*∗∗∗∗*^*P* < 0.0001 compared with the control group; ^#^*P* < 0.05, ^##^*P* < 0.01 compared with the CKD group).

Sirtuin3 is considered to be a key modulator of mitochondrial dynamics [14]. Regarding the expression of Sirtuin3 (Figure 4), it was observed that the untreated groups displayed a low-expression compared to the control group (*P* < 0.0001). The expression level of Sirtuin3 in the three treatment groups were as follows: HDD + PE > HDD > PE. The results suggested that HDD alone (*P* < 0.05) and HDD in combination with PE (*P* < 0.01) significantly increased Sirtuin3 expression in CKD rats.

### 3.5. Effects of PE and HDD on Mitochondrial Dynamics in CKD Rats

**Figure 5:**
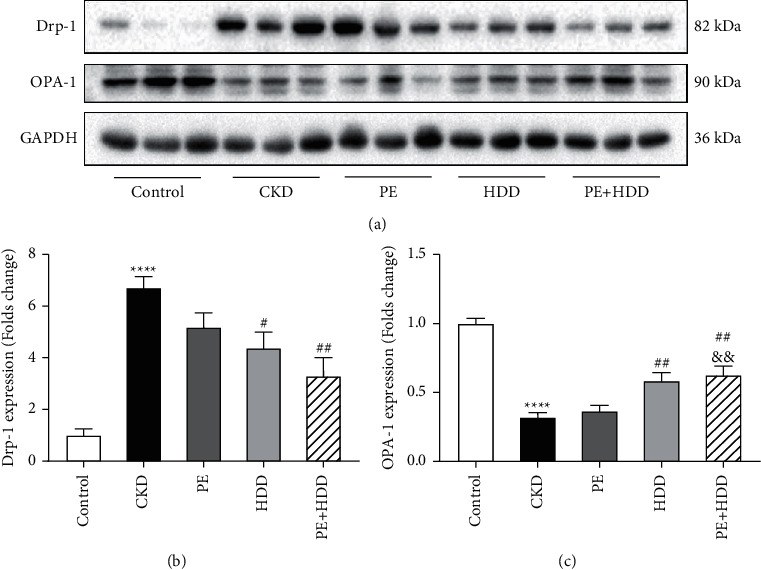
Effects of PE and HDD on mitochondrial dynamics in CKD rats. (a), Representative Western blot images of Drp-1 and OPA-1. (B–C), Quantitative analysis of protein expression of Drp-1 and OPA-1 protein, respectively. The data are expressed as the means ± SEM, *n* = 6 rats per group (^*∗∗∗∗*^*P* < 0.0001 compared with the control group; #*P* < 0.05, ##*P* < 0.01 compared with the CKD group; ^&&^*P* < 0.01 compared with the PE treatment group).

Mitochondrial fission and fusion constitute mitochondrial dynamics [13]. To further define the mitochondrial dynamics in CKD and regulatory effects of all treatments, protein levels of Drp-1 (mitochondrial fission protein) and OPA-1 (mitochondrial fusion protein) were evaluated respectively by Western blot (Figure 5). Compared with the control group, Drp-1 expression level was markedly upregulated in the CKD groups (*P* < 0.0001). Meanwhile, the expression of OPA-1 was reduced in the CKD group compared with the control group (*P* < 0.0001). This opposite trend of fission and fusion further confirmed the participant status of mitochondrial dynamics imbalance in the CKD progression. As shown in Figure 5(b), Drp-1 expression reduced by the combination treatment was the most significant (*P* < 0.01vs model). In addition, the expression of OPA-1 was raised by the HDD and combination treatment compared with the model group (*P* < 0.01) (Figure 5(c)). It is suggested that HDD or combine with PE treatment regulated mitochondrial dynamics in adenine-induced CKD rats through a bidirectional way. Moreover, the combination treatment exhibited a exceed effect of raising OPA-1 expression compared with PE treatment alone (*P* < 0.01).

### 3.6. Effects of PE and HDD on Mitochondrial Fragmentation in CKD Rats

**Figure 6:**
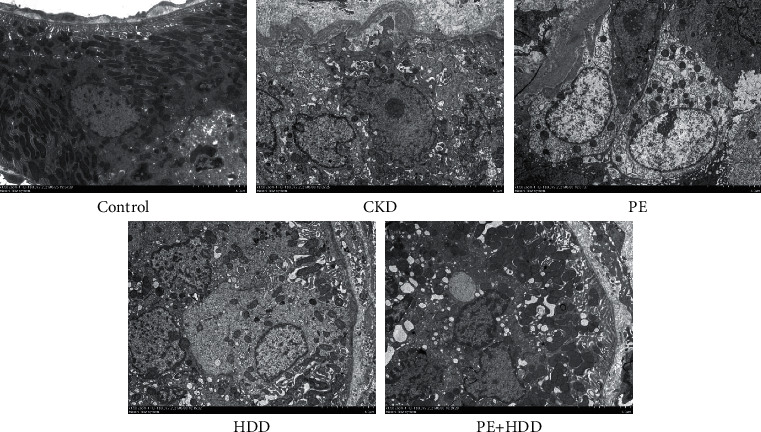
Effects of HDD and PE on mitochondrial fragmentation in CKD rats. Representative TEM pictures of mitochondrial morphology in all five groups.

As shown in Figure 6, mitochondria of CKD rats became split, punctate, fragmented when compared to the control group. In contrast, these mitochondrial morphological changes were partly restored by the HDD or in combined with PE treatment. These results indicated that the combination treatment prevented mitochondrial fragmentation in CKD rats.

## 4. Discussion

The present study demonstrated efficacy of PE combined with HDD in the treatment of adenine-induced CKD rats. Combination therapy exhibited a partial additive effect on renal dysfunctional protection and anti-fibrosis compared with either treatment alone. The mechanism might be related to Sirtuin3/mitochondrial dynamics pathway. Current study may afford more experimental evidences to the clinical application of RASIs in combination with TCM in CKD therapy.

CKD, associated with a high cost of care, is forecasted to become the fifth leading cause of death worldwide by 2040 [23]. Treatment with the RASIs is still the major clinical strategy after years of effort [4]. As such, further exploration of safety and efficacy treatment is necessary. Traditional Chinese medicine, due to its safety and efficacy, has been widely used for clinical application over thousands of years. Chinese herbal medicines combined with western medicine (such as RASIs) has become a complementary therapy for patients with CKD in decades [7, 24]. According to TCM theory, deficiency of Qi with blood stasis, refer to as “Qi-Xu-Xue-Yu”, is the common syndrome mode throughout various causes of CKDs. Therefore, reinforcing Qi and activating blood circulation is one of the basic principles of TCM applying for the CKD treatment [25]. Decoction is a conjunct form of Chinese herbal medicines. HDD is composed of Astragali Radix (Huang-qi), which conduced to reinforce Qi, and Salviae Miltiorrhizae Radix et Rhizoma (Dan-shen), which conduced to activate blood circulation. ﻿The present study showed the protective effect of HDD combined with PE in adenine-induced CKD rats. In CKD rats, significantly increased Scr and BUN concentrations were observed, which were consistent with the previous reports [26, 27]. Moreover, histological analysis exhibited distinct tubular atrophy and tubulointerstitial fibrosis in CKD rats. The apparently reversion of renal function reduces and histology by the combination treatment indicated that HDD in combination with PE could protect renal injury in adenine-induced CKD rats.

Mitochondria are extremely dynamics organelles, incessantly undergoing antagonistic two processes of fission and fusion, which composed the mitochondria dynamics [13]. Balanced mitochondria maintain mitochondria morphology, function, quality control and cellular homeostasis, while abnormal mitochondrial dynamics are linked with the various diseases, including cancer, neurodegenerative and cardia-cerebrovascular disease [28]. Excessed mitochondrial fission (moduated by Drp-1, Fis1, etc) was reported to promote tumorigenesis [29, 30]. Alternatively, impediment of mitochondrial fusion (ensured by OPA-1 and Mfn1/2) is associated peripheral neuropathy [31–33]. Emerging evidence has exhibited mitochondrial dynamics imbalance associated with the etiopathogenesis of renal diseases including diabetic nephropathy [34, 35] and acute kidney injury [36]. Accordingly, present study demonstrated that in the adenine-induced CKD rat model, compared with the decrease in OPA1 expression, Drp-1 expression was increased. In addition, this study presented that HDD in combination with PE had a certain trend of inhibitory effect on this imbalance by elevating the mitochondrial fusion and suppressing fission in adenine-induced CKD rats. Moreover, mitochondrial fragment in CKD model group was confirmed by transmission electron microscopy, whereas this morphologic anomaly significantly improved by the HDD and combination treatment.

Recent publications gave the prominent role of Sirtuin3 in various chronic disease, aging and cancer by mitochondrial dynamics modulation. Sirtuin3 is a major regulator of mitochondrial acetylome which located in the mitochondrial matrix and is mainly distributed in kidney, heart and other mitochondria-rich organs [14]. The initial investigation that Sirtuin3/Mitochondrial dynamics pathway occupied a role in the progression of chronic disease conditions was the discovery that Sirtuin3 directly targeting and deacetylated OPA1, thus controlled apoptotic from mitochondrial fusion [37]. Further studies demonstrated Sirtuin3-mitochondrial dynamics pathway has been supposed to become potential therapeutic target for cancer, neurodegenerative and autoimmune diseases [38]. Naia et al. demonstrated that Sirtuin3 overexpression favored mitochondrial dynamics balance by decreasing Drp1 and motivating mitochondrial fusion in Huntington's disease model [39]. Yi et al. found that activated Sirtuin3/OPA1 pathway remodeled the imbalance of mitochondrial dynamics in oxidative stress-induced melanocyte apoptosis in Vitiligo [40]. The latest study by Cheng et al. demonstrated that Sirtuin3KO mouse represented renal dysfunction and renal fibrosis exacerbation by regulating mitochondrial dynamics [41]. Taken together, these researches reveal that Sirtuin3-mitochondrial dynamics pathway has a pivotal role in various disease models. Enhancing Sirtuin3 activity or over expression improve mitochondrial dynamics, while Sirtuin3 deficiency leads to mitochondrial dynamics dysfunction, which accelerated disease progression. Accordingly, our research found that Sirtuin3 expression was downregulated in adenine-induced CKD rats, which was consistent with previous studies across diverse animal models. Conformably, transmission electron microscopy represented accumulated mitochondrial fragments, followed by dysfunction of mitochondrial dynamics and associated renal interstitial fibrosis in the development of adenine-induced CKD. In the cisplatin-induced AKI rat model, mitochondrial fission and ROS generation was reduced by Sirtuin3 overexpression, which contribute to prevent renal injury [17]. Renal ischemia-reperfusion injury was closely associated with the low expression of Sirtuin3, and the inhibited mitochondrial fusion was enhanced through the activated ERK/OPA1 signaling pathway [18]. Furthermore, our research found that the low expression of Sirtuin3 was improved by the combination treatment. In addition, the combination treatment reduced mitochondrial dynamics imbalance by upregulated OPA1/downregulated Drp-1 expression to varying degrees. However, the inherent mechanism of how Sirtuin3 modulate the mitochondrial dynamics in CKD demands further investigation. More work is need to identify the direct effects, primary targets and activities of Sirtuin3-dependent mitochondrial dynamics pathway, along with the potential therapy modulators in CKD.

## 5. Conclusion

In conclusion, the combination of PE and HDD attenuated adenine-induced CKD in rats. This protective effect was somewhat superior to PE alone. The underlying mechanism might be associated with the modulation of Sirtuin3/mitochondrial dynamics pathway.

## Data Availability

All data used and analyzed to support the current study are available from the corresponding author upon request.
